# Levels of certain tumor markers as differential factors between bilharzial and non-biharzial bladder cancer among Egyptian patients

**DOI:** 10.1186/1475-2867-11-8

**Published:** 2011-04-07

**Authors:** Nadia S Metwally, Sanaa A Ali, Azza M Mohamed, Hussein M Khaled, Samia A Ahmed

**Affiliations:** 1 Theraputic Chemistry Department, National Research Center, Dokki, Egypt; 2 National Cancer Institute, Cairo, Egypt

## Abstract

**Background/Objective:**

Bladder cancer is the commonest type of malignant tumors as a result of schistosomaisis which is a major healthy problem in many subtropical developing countries. The aim of this study is to comparatively elucidate the underlying biochemical tumor markers in schistosomal bladder cancer versus non-schistosomal bladder cancer when compared to normal healthy ones.

**Methods:**

This work was performed on tissue specimens from total 25 patients and serum samples from total 30 patients versus ten healthy individuals served as control. The investigated parameters in serum are: xanthine oxidase (XO), fructosamine, lactate dehydrogense (LDH), aspartate aminotransferase (AST), alanine aminotransferase (ALT), total proteins, essential and non- essential amino acids profile, hydroxyproline, total immunoglobulin E (IgE) and tumor necrosis factor alpha (TNF-*α*). In addition, the current investigation also extended to study some markers in tumor bladder tissues including, pyruvate kinase enzyme (PK), lactate dehydrogenase (LDH), aspartate aminotransferase (AST) and alanine aminotransferase (ALT).

**Results:**

Results showed that biharzial bladder cancer patients recored more significant elevation in serum XO, fructosamine, LDH, AST, ALT, hydroxyproline, IgE and TNF-*α *than in bladder cancer patients when compared to control ones. While, in tissues there were significant increase in PK, LDH, AST & ALT activities of schistosomal bladder cancer than in bladder cancer as compared to control healthy patients.

**Conclusions:**

It could be concluded that, bilharzial and non-bilharzial bladder cancer showed distinct biochemical profile of tumor development and progression which can be taken into consideration in diagnosis of bladder cancer.

## 1. Introduction

Bladder cancer is the second most common malignancy of the genitourinary system in males and females [1,2], accounting for 6% to 8% of all male malignancies and 2% to 3% of all female malignancies [3]. In Africa, squamous cell carcinoma of the bladder is greatly overrepresented among the fellaheen (peasant farmers) of Egypt and the Africans of Mozambique, Zimbabwe, and Zambia (formerly Rhodesia), all countries where *Schistosoma haematobium *is endemic. An age-standardized mortality rate for bladder cancer of 10.8 per 100,000 males places Egypt at the top of the list of the 54 countries providing data for the 1987 WHO database [4].

The most common type diagnosed in North and South America, Europe and Asia is transitional cell carcinoma, which is mainly non-schistosomal bladder tumors, followed by squamous cell carcinoma which is found more in geographical regions where schistosomiasis is prevalent [1]. One of the conditions leads to bladder cancer in Africa, the Middle East and Asia is schistosomiasis [5]. *Schistosoma haematobium *is the most predominant species in the Middle East, Asia, and Africa, and the most implicated in the schistosomal bladder tumors in these regions [6].

No prospective study measuring the risk of developing bladder cancer in infected and uninfected persons is yet available. Although differences in relative frequencies may reflect differences in risk, the interplay of other factors, such as geopolitical variations in case finding, can result in spurious differences and erroneous associations. If the postulated association is correct, one of several conditions must obtain: the worm (1) produces a carcinogen, (2) carries a virus, or (3) is cocarcinogenic to some other insult. In this case, there are many unanswered questions regarding geographic differences in vesical cancer observed where schistosomiasis is endemic. These range from whether there is geographic uniformity in the host's reaction to infection to whether other environmental variables (such as the bright food coloring used in the candy popular in the Nile delta) interact and are additionally responsible for vesical neoplasia [7].

There is some evidence suggesting that oxidative stress and bladder cancer are closely related [8,9]. Inherent oxidative stress may affect several functions in cancer cells or tumor tissue such as cell proliferation, promotion of mutations and genetic instability, alterations in cellular sensitivity to anticancer agents, invasion, and metastasis [10]. Hydroxyl radicals, peroxides and superoxides are reactive oxygen species (ROS) that are generated during everyday metabolic processes in a normal cell. ROS generated either endogenously (mitochondria, metabolic process, inflammation etc.) or from external sources play a vital role in regulating several biologic phenomena [11]. While increased ROS generation has traditionally been associated with tissue injury or damage of many different biomolecules including DNA, proteins, lipids and carbohydrates which are general manifestations of pathological conditions associated with infection, aging, mitochondrial DNA mutations and cellular proliferation [12-14]. Thus, excessive production of ROS or inadequacy in a normal cell's anti-oxidant defense system (or both) can cause the cell to experience oxidative stress and the increased ROS may play a broader role in cellular process associated with initiation and development of cancers [15]. In view of the above information, the objective of this study was undertaken to investigate some key findings relevant to ROS stress in bilharzial and non bilharzial bladder cancer compared to control healthy patients. The investigated parameters in serum including xanthine oxidase (XO) enzyme activity (as a marker of purine metabolism), fructosamine (as a marker of abnormal glucose metabolism), lactate dehydrogenase (as a maker of glucose metabolism), hydroxylproline (as a maker of liver fibrosis), as well as total serum immunoglobulin E (IgE), tumor necrosis factor-alpha (TNF-*α*) and liver function enzymes including aspartate and alanine aminotransferase (AST & ALT), total proteins and free amino acids were also measured in serum. The study was extended to investigate some biochemical parameters in tumor tissues isolated from bladder carcinoma patients including, pyruvate kinase (PK), AST, ALT and LDH enzymes.

## 2. Materials and methods

### 2.1 Clinical cases

All cases (55 individuals) were of Egyptian bladder carcinoma patients, attending to out clinic, Medicine Oncology and Pathology Departments - National Cancer Institute - Cairo University.

This work was performed on tissue specimens from total 25 patients and serum samples from total 30 patients. The patients were classified according to the clinical and pathological receptors into two subdivisions:

1. Having malignant carcinoma patients.

2. Having bilharzial bladder lesions.

Diagnoses were confirmed by microscopic studies, where the bladder cancer was sequamous cell carcinoma (SCC).

The tumors isolated surgically from a total of 25 individuals and divided into: 1) first group consists of 12 patients with bladder carcinoma [11 male, their ages ranged from 16-58 years and 1 female has 32 years old]. 2) Second group consists of 13 subjects having schistosomale infection [9 males, their ages ranged from 21-68 and 4 females, ranged from 34-59 years old].

The serum analysis was conducted on 30 patients, 15 bladder carcinoma [10 males (17-61 years old) and 5 females (26-39 years old] and 15 bilharzial bladder carcinoma [12 males (20-63 years old) and 3 females (18-35 years old)].

### 2.2. Control group

1. serum: ten healthy individuals were served as controls to all investigated serum groups.

2. tissue samples: The activities were measured in nighboring mucosa (unaffected tissues) to carcinoma tissues together with bilharzias bladder carcinoma tissues.

### 2.3. Gross Pathology

Tissue samples of bladder carcinoma, were subjected to both pathological and histological examinations in order to evaluate the criteria of diagnosis and diseases progress, at pathology department, NCI. The original cell microscopic slid were examined and the tumors were classified as squamous carcinoma which included grade I × II and adenocarcinoma cells including grade three (III).

### 2.4. Parameters Assays

#### For Serum tests

Serum was separated by centrifugation at 3000 rpm and subjected to the following biochemical tests: XO activity was determined by the reduction of nitro-blue tetrazolium (NBT) in the presence of xanthine, forming formazan. The enzyme activity was calculated using the extinction coefficient of reduced NBT(7.5 cm^2^/μmol at 540 nm) [16]. Fructosamine was determined according to the method of Parlin et al., [17] where fructosamine reduces NBT under alkaline condition and forms a purple-colored formazan with an absorption maximum at 530 nm. LDH was detected in serum using Beckman LDH kit. ASTand ALT were estimated using Stanbio kit produced by Stanbio Labs, Texas, USA. A total protein was determined by using the method of Lowry et al. [18]. Hydroxyproline content was measured using the method of Jamall et al. [19]. The level of total IgE was measured by ELISA at 450 nm and compared with known mouse IgE standard (BD PharMingen). TNF-α was quantified using a commercial ELISA kit (Endogen, Woburn, MA). The amino acids constituents of patient serum were determined using HPLC.Eppdrof -Germany-Lc3000 amino acid analyzer.

### 2.5. For tumors tissues

The tissues were free of necrotic debris, connective tissues and were immediately frozen after surgrey. Measurement of ASTand ALT activities in tumors tissue homogenates were carried out by colorimetric technique of Nabih et al. [20]. While LDH activity was assayed in the same homogenate by the method of Caboud and Wroblewski [21]. Pyruvate kinase was determined by using the method of Ibsen et al. [22].

### 2.6. Statistical analysis

All data obtained are expressed as the mean ± SD. Results were analyzed by a computerized statistical program. Values were compared by one-way ANOVA for multiple comparisons as the post hoc test. A P-value < 0.05 was considered to be statistically significant.

## 3. Results

Table (1) shows significant increase in serum XO enzyme activity, fructosamine, hydroxyproline, IgE, and TNF- α concentrations in bilharzial bladder cancer patients compared to non- bilharzial bladder cancer ones and control healthy patients. While data in Table (2) shows significant increase in serum enzymes including AST, ALT and LDH in both diseased patients groups compared to healthy cases. The results of total proteins reveals a moderate increase in bilharzial bladder patients as compared to control cases, but there is a non- significant decrease in total proteins in bladder cases compared to normal persons as shown in table (2). Table (3) shows highly significant increase in LDH, AST, ALT, and pyruvate kinase activities in both bilharzial and non bilharzial bladder cancer patients and the highest increase was observed in patients of grade three compared to healthy ones.

**Table 1:** Serum tumor markers in bilharzial and non-bilharzial bladder cancer patients compared to normal (unaffected) patients

Parameters	Control	Bilharzial bladder cancer patients	non-bilharzial bladder cancer patients	ANOVA P <
1. XO (μmol uric acid/min/ml) LSD	0.3 ± 0.05 (2,3)	2.77 ± 0.33 (1,3)	1.58 ± 0.23 (1,2)	P < 0.0001

2. Fructosamine (μmol/L) LSD	337.88 ± 34.4 (2,3)	1145.27 ± 146.16 (1,3)	742.3 ± 132.44 (1,2)	P < 0.0001

3. hydroxylproline (μg/ml) LSD	23.35 ± 2.83 (2,3)	78.56 ± 10.48 (1,3)	46.06 ± 4.28 (1,2)	P < 0.0001

4. IgE (IU/ml) LSD	44.37 ± 1.66 (2,3)	301.49 ± 14.04 (1,3)	269.0 ± 7.66 (1,2)	P < 0.0001

5. TNF-∝ (Pg/ml) LSD	7.8 ± 0.22 (2,3)	37.7 ± 1.61 (1,3)	24.6 ± 0.84 (1,2)	P < 0.0001

Data are represented as mean ± SD of 10 control and 30 patients (15 patients bilharzial and 15 non-bilharzial)

**Table 2:** Serum enzymes in bilharzial and non-bilharzial bladder cancerous patients in comparison to healthy persons:

Parameters	Grading progress	Healthy cases	Bilharzial bladder cancerous patients			non-bilharzial bladder cancerous patients		
			
			I	II	III	I	II	III
		
		n = 10	n = 4	n = 5	n = 6	n = 6	n = 4	n = 5
AST (U/L)	Mean ± S.D	28.4 ± 2.3	102.7 ± 6.9	120.6 ± 8.5	132.9 ± 10.1	85.3 ± 4.9	96.5 ± 9.3	107.3 ± 11.6
	P <	--	H.S	H.S	H.S	H.S	H.S	H.S

ALT (U/L)	Mean ± S.D	26.3 ± 1.8	82.2 ± 3.95	96.5 ± 6.1	111.1 ± 9.9	69.3 ± 6.5	83.3 ± 7.6	99.8 ± 6.7
	P<	--	H.S	H.S	H.S	H.S	H.S	H.S

LDH (U/L)	Mean ± S.D	139.9 ± 13.8	351.5 ± 20.2	434.7 ± 21.4	489.3 ± 37.6	334.4 ± 15.5	379.1 ± 14.2	408.6 ± 14.2
	P<	--	H.S	H.S	H.S	H.S	H.S	H.S

Total protein gm%	Mean ± S.D	7.7 ± 0.5	8.30 ± 0.6	8.40 ± 0.4	9.30 ± 0.3	7.40 ± 0.3	7.5 ± 0.4	7.80 + 0.5
	P <	--	S	S	S	N.S	N.S	N.S

H.S: P < 0.0005 N.S: P < 0.02

**Table 3:** Tumor marker enzymes in bilharzial and non-bilharzial carcinoma tissues compared to normal (unaffected) tissues:

Enzymes	Tissues types	Bilharzial bladder cancerous tissues n = 13						Bladder cancerous tissues n = 12					
		
		Unaffected (normal) tissues			Malignant tissues			Unaffected (normal) tissues			Malignant tissues		
	
	Grading	I	II	III	I	II	III	I	II	III	I	II	III
LDH	Mean ± SD	8.5 ± 0.33	8.8 ± 0.21	9.1 ± 0.12	15.4 ± 0.2	16.3 ± 0.21	17.3 ± 0.17	6.1 ± 0.14	6.9 ± 0.24	7.2 ± 0.17	10.8 ± 0.5	12.5 ± 0.29	13.5 ± 0.3
	P<	--	--	--	H.S	H.S	H.S	--	--	--	H.S	H.S	H.S

AST	Mean ± SD	1.74 ± 0.038	1.91 ± 0.01	2.06 ± 0.30	2.85 ± 0.05	3.44 ± 0.1	3.91 ± 0.13	1.61 ± 0.01	1.76 ± 0.04	1.93 ± 0.1	2.78 ± 0.03	3.19 ± 0.02	3.58 ± 0.13
	P<	-	-	--	H.S	H.S	H.S	--	--	--	H.S	H.S	H.S

ALT	Mean ± SD	4.69 ± 0.04	4.82 ± 0.05	4.98 ± 0.1	7.73 ± 0.3	7.96 ± 0.57	8.22 ± 0.4	4.59 ± 0.05	4.77 ± 0.13	4.86 ± 0.14	7.53 ± 0.02	7.82 ± 0.02	7.97 ± 0.03
	P<	--	--	--	H.S	H.S	H.S	-	--	--	H.S	H.S	H.S

PK	Mean ± SD	15.10 ± 3.70	15.21 ± 3.79	15.32 ± 3.81	36.1 ± 10.3	38.4 ± 12.6	42.3 ± 14.7	14.81 ± 3.12	14.97 ± 3.24	15.30 ± 3.51	24.90 ± 4.70	27.0 ± 10.6	30.1 ± 11.34
	P<	--	--	--	H.S	H.S	H.S	--	--	--	H.S	H.S	H.S

Activities were expressed as μ moles/mm/g tissue

H.S: Significant difference P <0 .0005

From our study, It is clear that all total amino acid levels significantly increased in bladder cancer patient with different percentage change except a-aminoadipic acid with -99.9%, glycine -35%, cystathionine -83.42%, tryptophan with -66% and carnosine with -41%. In bilharzial bladder cancer, Taurine, a-Aminoadipic acid, Glycine, a-Aminobutyric acid and Cystathionine were reduced with 8.06, 81.57, 94.92 and 49.0% respectively, while the other amino acids were elevated (tables 4,5).

**Table 4:** Essential amino acid fractions in different groups of bladder cancer and bilharzial bladder cancer patients

Parameters	Control	Bladder cancer	Bilharzial bladder cancer	% Change		
				
				(a)	(b)	(c)
Isoleucine	2.75 ± 0.390	5.27 ± 1.24	5.3 ± 0.89	+92	+52	+90

Leucine	5.36 ± 0.397	9.73 ± 2.88	9.99 ± 1.82	+81	+29	+86

Threonine	7.70 ± 1.175	9.78 ± 3.30	8.3 ± 2.63	+27	-37	+8

Valine	3.15 ± 0.83	10.28 ± 4.43	11.6 ± 2.32	+226	+51	+267

Phenylalanine	3.12 ± 0.46	5.07 ± 1.07	4.7 ± 0.78	+62	+31	+51

1-Methyl-Histidine	5.22 ± 0.58	7.48 ± 1.167	6.7 ± 2.27	+43	+36	+29

Lysine	8.56 ± 1.43	16.59 ± 3.98	16.9 ± 3.22	+94	+75	+97

Trytophan	1.34 ± 0.39	0.000 ± 0.00	1.5 ± 1.35	-100	-66	+15

Carnosine	2.84 ± 0.58	1.18 ± 0.62	2.1 ± 0.71	-58	-41	-26

Cystathionine	0.923 +0.59	0.153+0.14	0.47 +0.99	-83	-84	-49

• Data are means ± S.D. of five patients in each group.

• Total amino acid is expressed as g/L serum.

• (a, b and c) percentage change as compared with control.

**Table 5:** Non-essential free amino acid fractions in different groups of bladder cancer and bilharzial bladder cancer patients

Parameters	Control	Bladder cancer	Bilharzial bladder cancer	% Change		
				
				(a)	(b)	(c)
Asparatic acid	2.61 ± 1.07	5.399 ± 2.230	14.141 ± 10.35	+106.78	+29	+442

Proline	4.46 ± 0.24	14.84 ± 5.37	10.227 ± 3.13	+232.5	+177	+129

Glycine	14.14 ± 2.72	9.19 ± 1.22	6.917 ± 4.55	-35	-99.98	-51

Alanine	1.09 ± 0.23	19.75 ± 7.43	14.421 ± 3.34	+1703	+1457	+1216

Serine	7.98 ± 0.81	12.03 ± 3.15	8.977 ± 2.20	+51	+19	+13

Glutamic acid	31.38 ± 3.16	35.32 ± 4.64	44.53 ± 14.03	+13	+33	+42

Tyrosine	4.23 ± 0.35	5.92 ± 1.91	6.455 ± 0.79	+40	+40	+53

NH4+	4.23 ± 1.16	9.43 ± 1.94	5.955 ± 2.74	+123	+11	+41

Phosphoserin	0.19 ± 0.06	0.24 ± 0.086	0.280 ± 0.21	+27	-1.1	+49

Taurin	3.35 ± 0.49	6.53 ± 1.69	3.075 ± 0.23	+95	-18	-8

Urea	76.5 ± 9.86	88.28 ± 7.24	111.95 ± 9.32	+15	+ 5	+46

Cystine	3.22 ± 0.57	3.99 ± 0.81	3.884 ± 1.15	+24	-37	+21

Citrulline	0.96 ± 0.41	1.51 ± 0.36	1.198 ± 0.41	+147	-42	+24

Ornithine	4.396 ± 0.71	11.114 ± 3.42	5.467 ± 0.81	+153	+75	+24

Arginine	3.58 ± 0.71	7.72 ± 1.23	27.3 ± 3.01	+115	+289	+663

a-Aminobutyric acid	0.0197 ± 0.01	0.188 ± 0.32	0.0010 ± 0.001	+854	+28	-95

a-Aminoadipic acid	1.530 ± 0.29	0.0012 ± 0.002	0.282 ± 0.261	-99.9	-100	-82

• Data are means ± S.D. of five patients in each group.

• Total amino acid is expressed as g/L serum.

• (a, b and c) percentage change as compared with control.

## 4. Discussion

Oxidative stress induces a cellular redox imbalance that has been found to be present in various cancer cells as compared with normal cells, the redox imbalance thus may be related to oncogenic stimulation [23]. The disturbance of the pro-oxidant/antioxidant balance, resulting from increased free radical production, antioxidant enzyme inactivation, and excessive antioxidant consumption, is the causative factor in oxidative damage [24]. There are potentially different types of DNA lesions resulting from ROS and reactive nitrogen species, which could be mutagenic and involved in the etiology of cancer [25].

Xanthine oxidase (XO: EC 1.2.3.2) is an enzyme playing a part in purine metabolism. It catalyzes the conversion reactions of hypoxanthine to xanthine and xanthine to uric acid, the last reaction in the purine catabolism, with byproduct of toxic superoxide radical. In this regard, it is a key enzyme between purine and free radical metabolism. It was reported that XO is an endogenous source of ROS and reactive nitrogen species (RNS) that can induce oxidative stress and inflect tissue injury [26,27]. There is growing evidence that superoxide radicals generated by XO are primarily responsible for the cellular deterioration associated with several conditions [28].

The data reported in the literature on oxidant, antioxidant molecule, and enzymes in different human cancer types are controversial. Our findings show significant increase in XO activity of bilharzial bladder cancer and non-bilharzial bladder cancer patients compared to normal healthy ones. Kokoglu et al. [29] found higher XO activity in tumoral brain tissues and suggested that the levels of XO in brain tissues could be used as a biochemical marker for the differentiation of tumoral tissues from normal ones. Our findings are consistent with Kaynar et al. [30] who found an increase in XO activity in patients with small cell and non-small cell lung cancer. Also, Ozturk et al. [31] observed a significant increase in XO activity in patients with colorectal cancer compared to control group. The authors attributed this increase in this enzyme to an unbalancement alteration in oxidant-antioxidant status due to the cancer process. In addition, Gülec et al. [28] found that XO activity increased in bladder cancer patients suggested that oxidative stress might be increased in cancerous changes and process, and may affect the course of the disease. The burst of XO-mediated free oxygen radical generation in the cancerous tissue can be triggered by a large increase in substrate formation which occurs secondary to the rapid turn-our of adenine nucleotides during cancer process. On the other hand, as proposed by Durak et al. [32] high XO activity may be an attempt to lower salvage pathway activity for purines, which is vital for rapid DNA synthesis in cancerous bladder tissue. Moreover, Mohamed et al. [33] found a significant increase in XO activity in mice infected with *S. mansoni *parasite.

Given the evidence that supports a relationship between abnormal glucose metabolism and cancer risk, serum fructosamine, one of the markers of abnormal glucose metabolism, is a glycated protein resulting from spontaneous, non enzymatic condensation of glucose and proteins, produces an unstable ketoamine, which is generally referred to as fructosamine due to its structural similarities to fructose [34,35]. As albumin is the most abundant protein in serum and contains multiple lysine residues, measurement of fructosamine is mainly the determination of glycated albumin [36]. This non enzymatic modification of protein is considered by several authors as a possible common mechanism involved in the progression of many pathological conditions such as myocardial and renal fibrosis, colorectal adenoma and chronic renal failure [35,37].

The current investigation revealed that there was a highly significant increase in fructosamine level in bilharzial bladder cancer and non-bilharzial bladder cancer patients compared to normal ones. This data is in line with Misciagna et al. [35,38] who reported that fructosamine is associated with colorectal adenoma. Also, Platek et al. [39] observed a 60% risk increase for breast cancer in women with high levels of fructosamine, suggesting that fructosamine, as an indicator of glucose consumption, may be a predictor of breast cancer. Recently, Pasanisi et al. [40] found that fructosamine was not associated with recurrence of breast cancer. Mohamed et al. [33] reported an increase in serum fructosamine of *S. mansoni *infected mice, this results was in accordance with our result in case of bilharzial baldder cancer patients. Our results are important because this is the first study investigating serum fructosamine in relation to bladder cancer and may be used as a useful marker for bladder carcinoma risk.

Malignant tumors inhabit a complex carbohydrate metabolism which differs from that of non-neoplastic cells with two main paradigms: 1) malignant cells produce large amounts of lactate even in the presence of sufficient oxygen for aerobic glycolysis. 2) intermediates of the tricarboxylic acid cycle (TCA) are used for fatty, amino and nucleic acid synthesis [41,42]. Thus, the extensive glucose uptake of cancer cells is needed not only for energy supply but also to provide the components for cellular growth and a high amount of reducing equivalents such as NADPH[41,42]. To accomplish this, high levels of pyruvate are needed which can be introduced either into the TCA, converted into acetyl-COA or degraded to lactate by LDH[41-43]. By degradation of pyruvate to lactate by LDH, the pool of reductive equivalents on the one hand and the availability of citric acid cycle intermediates for fatty and amino acid synthesis on the other hand is raised. The overexpression of LDH_5 _in tumor cells supports this theory of a glucose metabolism optimized for cellular growth within malignant tumors.

Results in the present study showed a highly significant increase in LDH activity in both serum and bladder carcinoma tissues of bilharzial bladder and non-bilharzial bladder patients compared to healthy ones. The highest significance was observed in grade III of the disease. These results are in harmony with Kayser et al. [44] who found that immunohistochemical analysis for LDH_5 _expression in non-small cell lung cancer (NSCLC) is specific to differentiate malignant neoplasia from healthy lung tissue. The authors reported that LDH_5 _overexpression in cancer cells induces an upregulated glycolytic metabolism and induced dependence on the presence of oxygen. In addition, Wu et al. [45] found that the level of LDH was significantly higher in patients with liver metastasis of colorectal cancer than those without liver metastasis.

Another glycolytic enzyme was also estimated in the present study, which is pyruvate kinase enzyme, catalyzing the conversion of phosphoenol pyruvate into pyruvate. Our results revealed significant increase in the activity of PK enzyme in bilharzial and non-bilharzial bladder tissues when compared to the unaffected normal ones. In accordance with our data Shonk et al. [46] studied the activities of different glycolytic enzymes in carcinoma of rectum and colon and in the corresponding non-malignant counterparts. They observed that glycolytic enzyme activities of the neoplastic tissues of rectum and colon are much higher than the activites in the corresponding normal rectum and colon. Also, Ramo Rao [47] stated that the glycolytic enzyme pyruvate kinase requires potassium for its maximum activity i.e., the enzyme activity correlates with potassium concentration in tissues or blood. In support, Reddy et al. [48] expected that the levels of potassium which may be a cofactor for pyruvate kinase should be higher in carcinoma tissues. Recently, Rautray et al. [49] observed that a potassium level in the carcinoma blood of gallbladder is lower than those in the normal blood samples. Potassium is a major intracellular cation which is also excreted in gastrointestinal tract, sativa, gastric juice, bile, pancreatic and intestinal juices, its deficiency causes acidosis, renal damage and cardiac arrest.

The metabolism of healthy and functionally active cells is optimized for productivity in terms of fulfilling their duty to synthesize, degrade, transport or contract. In contrast to this, malignant tumor cells do not meet these demands but only strive for cellular growth and mitosis [44]. According to this theory high amounts of ATP as demanded within healthy cells are not only not required but act contra - productive for the synthesis of basic elements for cellular growth such as proteins, nucleic acids and fatty acids. Protein is a critical reservoir of metabolic fuel and may become seriously depleted during tumor growth [50]. The metabolism of protein and amino acids in cancer patients is closely linked to glucose metabolism and is regulated by a number of the same hormones and metabolites [51].

Our findings in the current study showed a slight increase (P < .025) in total protein concentration in serum of bilharzial bladder cancerous patients compared to healthy ones while, there was non-significant decrease in serum total protein of bladder cancer patients when compared to healthy ones. These results are in harmony with Lai et al. [50] who found that in cancer patients, increased hepatic protein synthesis and gluconeogenesis might lead to a slight decrease in plasma free amino acids levels and a slight increase in plasma protein levels. On contrary to our results, Rossi Fancelli et al. [52] reported that whole - body protein breakdown has been demonstrated to increase in cancer patients and correlated with gluconeogenesis in malnourished cancer patients.

On the other hand, Sauer and Dauchy, [53] found that fructose - 1, 6 - disphosphatase, the enzyme involved in the conversion of pyruvate to glucose is elevated in the liver, suggesting that liver protein is utilized for gluconeogenesis in cancer patients. Recently Kojima et al. [54] detected an elevated proteins in serum of gastric cancer patients with peritoneal dissemination compared with serum samples from patients with gastric cancer in general.

On the contrary of our results, Alexandrakis et al. [55] found a significant reduction in serum total protein in malignant neoplasms and cirrhotics. Also, Haitel et al. [56] stated that transitional cell carcinoma and squamous cell carcinoma of the bladder differ in terms of protein expression and prognosis. In addition Ali et al. [57] found a significant reduction in serum total protein content in bladder cancer, and bilharzial bladder cancer patients compared to the control healthy group and the authors recorded that the decrease was more in bilharzial bladder cancer group than the two other groups.

The data obtained in the current study of diagnostic marker enzymes revealed a marked increase in the activities of aspartate and alanine aminotransferases in serum and tissues of bilharzial bladder cancer and non-bilharzial bladder cancer versus healthy controls and the highest levels in activities of enzymes were obvious in grade III of the bladder cancer.

The increased of AST and ATL levels were more specified extra cellular release and decreased protein content. Several reports confirmed the elevation in AST and ALT activities as a result of *S.mansoni *infection which may reflect a decrease in hepatic cells, where Salah et al. [58] and Ahmed [59] indicated the decrease in hepatic enzymes activities may be due to the release of the enzymes from the necrotic tissue or due to increased cell membrane permiability as a result of relative anoxia and irritation by toxic or metabolic wastes of the worms.

The extra cellular release of the transaminases leads to their increase in serum [60], while the reduction of enzymes activities relative to lower liver protein content was either due to their release in the bloodstream or due to their decrease synthesis, since transaminases can serve as an index of metabolic aerobity degree, and AST can provide Krebs cycle intermediates, while ALT can be correlated with lactate production [61]. The authors also reported that the elaborated ROS causing damage of cellular membrane, and hence, AST and ALT are sub-cellular enzymes localized in mitochondria and cytoplasm, their release to the bloodstream was increased and their synthesis at the same time decreased.

In accordance with our findings, Ali et al. [57] who recorded more significant elevation values of AST, ALT in bilharzial and non bilharzial bladder cancer patients compared to normal group. Recently, Wu et al. [45] found that the levels of ALT and AST were significantly higher in patiens wih liver metastasis of colorectal cancer than in those without liver metastasis.

The amino acids can be classified into two major categories. The essential amino acids are obtained solely from the diet, whereas the body can, under some circumstances, produce non-essential amino acids from other sources. In certain cancers the amino acid profile yields useful data about the disease from which treatment approaches may be better assessed. Colorectal cancer patients exhibit a characteristic amino acid profile: significantly lower taurine, glutamine, valine, and tyrosine in plasma; significantly lower taurine, glutamic acid, methionine and ornithine intracellularly; and elevated valine, isoleucine, leucine, tyrosine, and phenylalanine intracellularly. Obtaining the amino acid profile may help to construct an appropriate dietary manipulation program aimed at modifying intracellular amino acid concentration[62]. Likewise, squamous cell carcinoma of the head and neck exhibit a profile that is marked by decreased taurine, alanine, asparagine, aspartic acid, glycine, histidine, ornithine, phenylalanine, serine, and threonine, with a marked increase in levels of cystine. These features are noted regardless of the stage of the cancer and nutritional status. Thus, serum amino acid levels in serum and other tissues may become a useful cancer marker cancer and provide valuable prognostic information [63].

Remarkable difference in amino acid profiles between the colorectal cancer patients and the control subjects is found in this study and most of the differences are statistically significant in respect of non-essential amino acids. The difference in essential amino acids concentration was noticeably insignificant except for methionine and theronine. Furthermore, some studies showed that the concentration of amino acids in subjects might be correlated with the same diseases[64].

The current investigation showed a significant increase in hydroxyproline level in serum of bilharzial bladder cancer and non-bilharzial bladder cancer versus control healthy ones. The increase was more in bilharzial bladder cancer group than the two other groups. In accordance with our data, Proenza et al. [65] demonstrated an increased levels of hydroxyproline in breast cancer. Also, Mohamed et al. [33] reported a marked increase in the level of liver hydroxyproline in infected mice versus normal healthy ones. Because collagen is the main element of extracelleular matrix proteins, hydroxyproline, is an amino acid characteristic of collagen metabolism, used as a marker to express the extent of liver fibrosis, as it is the major alteration associated with morbidity [66]. Similar result was obtained by some authors who emphasized that elevated liver hydroxyproline content was associated with *S. mansoni *infection [67,68] and this may be attributed to that *S. mansoni *egg granulomas contain factors reposible for the elevation of free L-hydroxyproline content in the fibrotic liver [69].

Although IgE is well known for its role in atopic disease and parasitic infections, new evidence suggests that IgE is a pleiotropic Ig molecule with many more functions. Previous studies have shown that IgE possesses antiviral activity, in that IgE anti-HIV-1 antibodies were able to elicit viral clearance and killing through inhibiting virus production [70].

Data in the present work showed a significant increase in IgE level in bilharzial and non-bilharzial bladder cancer compared to normal group. It is well established that helminthes infestation induces a very high level of serum IgE which is present at both the initial and later stages of schistosomiasis [71]. In western countries where the incidence of parasitic disease is very low, a raised IgE may also occur in some patients with cancer [72].

In accordance with our findings, Ottesen et al. [73] indicated that IgE is directed mainly against egg antigen in patients with acute bilharziasis, but it is directed equally to cercarial, egg and adult worm antigens in patients with chronic infestation. In addition, Pidcock et al. [74] reported that the well established increase in IgE level in patients with schistosomiasis, was also found in bilharzial bladder cancer, indicating that humeral immunity persists in cancer - bearing patients. Patients with cancer not associated with parasitic infestation also had significant increase in their serum level of IgE when compared to healthy Egyptian controls, but 41% of these non-bladder cancer patients showed IgE responses to previous parasitic infestations suggesting that only immunological response to cancer would be on the background of a variable non-specific increase of IgE.

IgE was reported to have the major role in mast cells stimulation which has a central role in the induction of chronic inflammation [75] and the progression of hepatic fibrosis by producing fibrogenic inflammatory mediators as well as the components of the extracellular matrix proteins (ECMPs) [76,77]. Elevated IgE levels have been reported in Hodgkin's lymphoma (HL) and advanced stage of HL disease has also been found to be correlated with elevated IgE [78].

It has been postulated that the association between HL and high serum IgE levels is linked to suppressor lymphocyte dysfunction (CD8+) and is distinct from the increase in allergen - specific IgE, which is associated with atopy [79]. Recently, Fu et al. [80] reported the presence of elevated IgE and its receptor SCD23 in serum obtained from pancreatic cancer patients versus controls, whereas other Ig isotypes (IgG, IgM, IgA) did not differ between patient and control populations. Also, Chang et al. [81] reported that high maternal IgE levels were positively associated with childhood leukemia, suggesting that maternal immune function may play a crucial role in the etiology of childhood leukemia.

The current work was also extended to study the inflammatory cytokine, tumor necrosis factor - alpha (TNF-*α*) in bilharzial and non-bilharzial bladder cancer patients. The data revealed a significant increase in TNF-*α *level in bilharzial and non-bilharzial bladder cancer patients versus normal controls. These results are in harmony with Wahl and Kleinman, [82] who found an increase in TNF-*α *in prostate cancer patients and they attributed this increase in TNF-*α *level to the increase of the inducible nitric oxide synthase (iNOS) which is one of the major responses of inflammatory component in a neoplastic transformation. As a result nitric oxide (NO) species have been implicated not only in direct damage to cellular components like DNA and proteins, but can also cause changes to the antioxidant defense of a cell along with increased ROS and could be one of the driving factors for promoting prostate cancer [83]. Therefore, in addition to ROS generated by inflammatory cells, uncontrolled tumor cell proliferation in an environment rich in growth factors, activated stroma and tumor associated neo-vascualization, can potentiate and/or promote the development of prostate cancer [84].

Beside, it was reported that TNF-*α *essentially functions as a trophic factor for maintaining adult schistosome viability, it is expressed during egg deposition and has a crucial role in the modulation of granulomatous reaction induced by the eggs [85]. Torben and Hailu [86] stated that increased level of this inflammatory cytokine after egg excretion may be an indication of its effect in complications of schistosomiasis, it capable of inducing tissue injury and fibrosis through inducing ROS production, lipid peroxidation [87], collagen synthesis other fibrogenic risk factors [88].

In summary, our results add to exicisting evidence by showing the complexity of the association of bilharzias and bladder cancer. This association seems to be dependent on the repeated infestion of the bladder with *S.haematobium *parasite. This study compared the profile of the studied biochemical parameters among bilharzial and non bilharzial bladder cancer and normal control subjects. This work is believed to highlight the essential biochemical markers that can be important candidates for bladder cancer diagnosis. In this study, we found that serum fructosamine, as an indicator of glucose consumption, may be a predictor of bladder cancer, also the amino acid, hydroxylproline, as an indicator of liver fibrosis, may be a predictor of bladder cancer risk, in addition, IgE and TNF-*α *are also may be used as a new immunological markers of bladder cancer.

## Conclusion

As a conclusion, increased of all previously mentioned markers in both serum and tissues of patients might be a potentially important findings as an additional diagnostic biochemical tools for bladder cancer.

## Authors' contributions

NM, SA, AM and SA carried out the experiments, drafted the manuscript and Participated in the design of the study, HK supplying us with the patient samples that used in this work from National Cancer Institute, Cairo, Egypt. All authors have read and approved the final manuscript.

## Competing interests

The authors declare that they have no competing interests.
